# HMGB2 is a novel adipogenic factor that regulates ectopic fat infiltration in skeletal muscles

**DOI:** 10.1038/s41598-018-28023-7

**Published:** 2018-06-25

**Authors:** Deokcheol Lee, Noboru Taniguchi, Katsuaki Sato, Narantsog Choijookhuu, Yoshitaka Hishikawa, Hiroaki Kataoka, Hidetaka Morinaga, Martin Lotz, Etsuo Chosa

**Affiliations:** 1 Department of Orthopaedic Surgery, University of Miyazaki, 5200 Kihara, Kiyotake, Miyazaki, 889-1692 Japan; 2 Institute of Medical Science, Tokyo Medical University, 6-1-1 Shinjuku, Shinjuku-ku, Tokyo, 160-8402 Japan; 3 Division of Immunology, Department of Infectious Diseases, University of Miyazaki, 5200 Kihara, Kiyotake, Miyazaki, 889-1692 Japan; 4 Department of Anatomy, Histochemistry and Cell Biology, University of Miyazaki, 5200 Kihara, Kiyotake, Miyazaki, 889-1692 Japan; 5 Section of Oncopathology and Regenerative Biology, Department of Pathology, University of Miyazaki, 5200 Kihara, Kiyotake, Miyazaki, 889-1692 Japan; 6 Department of Internal Medicine and Bioregulatory Science, Graduate School of Medical Sciences, Kyushu University, 3-1-1 Maidashi, Higashi, Fukuoka, 812-8582 Japan; 7 Department of Molecular Medicine, The Scripps Research Institute, 10550 North Torrey Pines Road, La Jolla, CA 92037 USA

**Keywords:** Experimental models of disease, Molecular medicine

## Abstract

Although various surgical procedures have been developed for chronic rotator cuff tear repair, the re-tear rate remains high with severe fat infiltration. However, little is known about the molecular regulation of this process. Mesenchymal stem cells (MSCs) in the intra-muscular space are origin of ectopic fat cells in skeletal muscle. We have previously shown that high-mobility group box 2 (HMGB2), which is a nuclear protein commonly associated with mesenchymal differentiation, is involved in the early articular cartilage degeneration. In this study, we addressed the role of HMGB2 in adipogenesis of MSCs and fat infiltration into skeletal muscles. HMGB2 was highly expressed in undifferentiated MSCs and co-localized with platelet-derived growth factor receptor α (PDGFRA) known as an MSC-specific marker, while their expressions were decreased during adipocytic differentiation. Under the deficiency of HMGB2, the expressions of adipogenesis-related molecules were reduced, and adipogenic differentiation is substantially impaired in MSCs. Moreover, HMGB2^+^ cells were generated in the muscle belly of rat supraspinatus muscles after rotator cuff transection, and some of these cells expressed PDGFRA in intra-muscular spaces. Thus, our findings suggest that the enhance expression of HMGB2 induces the adipogenesis of MSCs and the fat infiltration into skeletal muscles through the cascade of HMGB2-PDGFRA.

## Introduction

Recent studies have revealed a relationship between the severity of fat infiltration into skeletal muscles, as observed in ruptured rotator cuffs, and clinical outcomes^1,2^. Although various surgical procedures have been developed to restore cuff integrity, including bone marrow stimulation^3^, the re-tear rate remains high for chronic rotator cuff tear (RCT), which is associated with severe fat infiltration^2^. The emergence of adipocytes in muscles might be attributed to mesenchymal stem cells (MSCs)^4^, and the loss of mechanical stretch may initiate adipogenic pathways in pluripotent stem cell and precursor cell populations within the muscle, leading to the phenotypic changes observed with fat infiltration^4^.

Recently, platelet-derived growth factor receptor α (PDGFRA)-positive mesenchymal progenitors have been reported to contribute to adipogenesis in skeletal muscle and to be responsible for ectopic fat cell formation in skeletal muscle under pathological conditions^5–7^. In addition, PDGFRA is an MSC-specific cell surface marker, along with stem cell antigen-1 (Sca-1)^8,9^, which is widely accepted as a marker of stem cell enrichment in tissues^10^.

HMGB2 is a member of the high-mobility group box (HMGB) protein family, which also includes ubiquitous HMGB1 and embryo-specific HMGB3. These three proteins are homologous for 80% identity at the amino acid level and characterized by two basic HMG box domains followed by a long acidic tail^11^. As nuclear proteins, HMGB1 and HMGB2 regulate various cellular activities, including transcription, DNA replication, and repair^12^. They bind to transcription factors, such as steroid hormone receptors, p53, p73, LEF1, and Runx2^13–16^, and enhance the transcriptional and recombination activities of their partner proteins. HMGB2 is detected at high levels in human MSCs, and its expression decreases during chondrogenic and osteogenic differentiation^16^. HMGB2 also regulates various other differentiation programs, including erythropoiesis, spermatogenesis, neurogenesis and myogenesis^17–20^. However, the function of HMGB2 in adipogenesis has not yet been elucidated.

The present study aimed to clarify the role of HMGB2 in MSC adipogenesis. We also investigated its role in fat infiltration into skeletal muscles following rotator cuff tear and the relationship between HMGB2 and PDGFRA during this process.

## Results

### Microarray analysis of adipogenesis-related markers in wild-type (WT) and *Hmgb2*^−/−^ MSCs

Microarray analysis of wild-type (WT) and *Hmgb2*^−/−^ MSCs identified 426 differentially expressed transcripts; 124 transcripts were decreased and 302 transcripts were increased in *Hmgb2*^−/−^ MSCs as compared with WT cells. The transcriptional expressions of adipogenesis-related genes, including *Ebf1*^21^, *Lifr*^22^, *Lpl*, *Fabp4*, *Ppargc1a*, *Pparg*, and *Cebpa*^23–26^ were markedly decreased in *Hmgb2*^−/−^ MSCs when compared with WT cells (Table 1). Furthermore, the pathway analysis of 426 differentially expressed genes with GeneSpring demonstrated that *Hmgb2* deficiency in MSCs was associated with the remarkable suppression of adipogenesis gene pathway and the PPAR signaling pathway as well as white fat cell differentiation pathway (Table 2). In contrast, the genes involved in the Wnt signaling pathway, which reportedly inhibits the adipogenesis of MSCs^27^, were significantly increased in *Hmgb2*^−/−^ MSCs (Table 2).

**Table 1:** Adipogenesis-related genes including *Pparg* and *Cebpa* were suppressed in *Hmgb2*^−/−^ MSCs.

Gene symbol	*Hmgb2*^−/−^ 1	*Hmgb2*^−/−^ 2	WT 1	WT 2	ave (*Hmgb2*^−/−^)/ave (WT)
*Hmgb2*	32.8	52.4	1,406.4	2,398.2	0.02
*Lpl*	26.8	30.4	1,218.0	888.2	0.03
*Fabp4*	65.1	135.9	435.2	1,033.7	0.14
*Pparg*	214.1	231.4	869.7	487.9	0.33
*Ebf1*	114.9	43.6	269.4	119.1	0.41
*Lifr*	54.6	82.3	218.3	112.7	0.41
*Ppargc1a*	33.9	34.1	110.7	46.4	0.43
*Cebpa*	40.6	38.3	70.4	96.9	0.47

The list of genes whose expression levels were less than half in *Hmgb2*^−/−^ MSCs compared to in WT MSCs.

**Table 2:** Pathway analysis of genes that were considerably inhibited or activated in *Hmgb2*^−/−^ MSCs.

Inhibited pathways in *Hmgb2*^−/−^ MSCs	p-value	Activated pathways in *Hmgb2*^−/−^ MSCs	p-value
Adipogenesis genes	3.55E-06	Focal Adhesion	5.22E-09
White fat cell differentiation	4.84E-04	XPodNet-protein-protein interactions in the podocyte expanded by STRING	5.52E-09
Retinol metabolism	8.70E-04		
Chemokine signaling pathway	0.00179	PodNet-protein protein interactions in the podocyte	8.44E-09
Selenium micronutrient network	0.00541	Primary focal segmental glomerulosclerosis FSGS	1.12E-07
PPAR signaling pathway	0.00655	Focal adhesion-PI3K-Akt-mTOR-signaling pathway	3.05E-07
Oxidative stress	0.00797	Matrix metalloproteinases	1.01E-06
Prostaglandin synthesis and regulation	0.00971	Integrin-mediated cell adhesion	2.43E-05
Ovarian infertility genes	0.00971	IL-3 signaling pathway	2.43E-05
Focal adhesions	0.0116	p53 signaling	0.0013
Nuclear receptors	0.0144	TGF beta signaling pathway	0.00338
Tryptophan metabolism	0.0182	Osteoblast	0.00595
Selenium metabolism selenoproteins	0.0215	miRNAs involved in the DNA damage response	0.00595
S1P pathways and spinal cord injury	0.0285	Complement and coagulation cascades	0.006
Metapathway biotransformation	0.029	Alpha6-beta4 Integrin Signaling pathway	0.00791
		MAPK signaling pathway	0.015
		IL-7 signaling pathway	0.0154
		ErbB signaling pathway	0.0173
		Delta-Notch signaling pathway	0.018
		EGFR1 signaling pathway	0.0185
		PluriNetWork	0.0228
		Hypertrophy model	0.0232
		Regulation of actin cytoskeleton	0.0346
		Endochondral ossification	0.0376
		MAPK signaling pathway	0.0408
		Wnt signaling pathway NetPath	0.0413
		miRNA regulation of DNA damage response	0.0423
		Kit receptor signaling pathway	0.0456
		TFs regulate miRNAs related to cardiac hypertrophy	0.0467
		IL-5 signaling pathway	0.0491
		Inflammatory response pathway	0.0492

Pathway analysis was performed for the 426 differentially expressed genes between WT and *Hmgb2*^−/−^ MSCs. In *Hmgb2*^−/−^ MSCs, adipogenesis-related pathways, such as the PPAR signaling pathway, and white fat cell differentiation-related pathways were significantly suppressed, whereas the Wnt signaling pathway was significantly enhanced.

### HMGB2 Expression in MSCs during adipogenic differentiation

**Figure 1:**
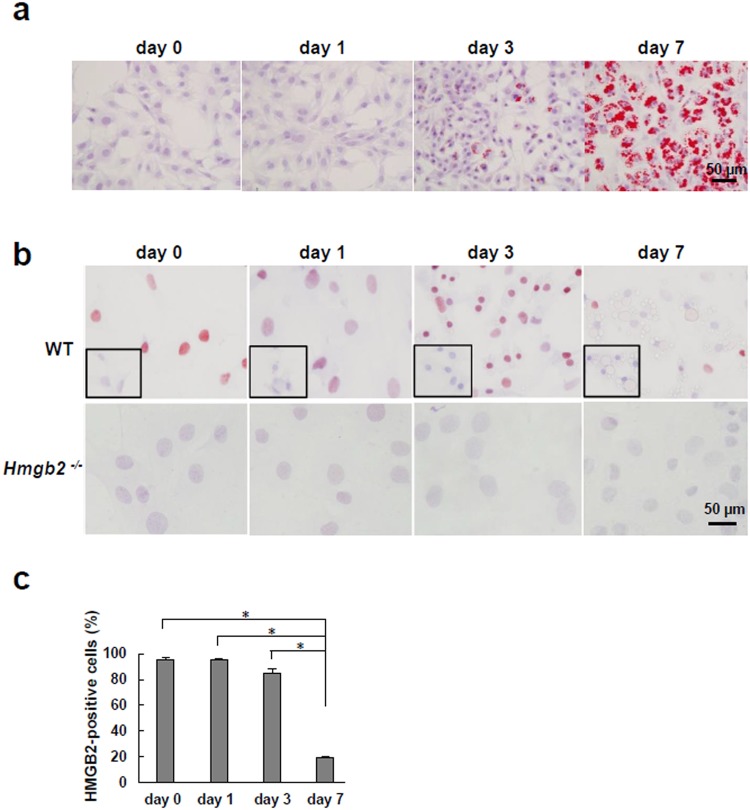
The expression of HMGB2, which was highly expressed in undifferentiated MSCs, decreased during adipocyte maturation. (**a**) Oil-red O staining of WT MSCs during adipogenesis (original magnification, x400, 0.237 mm^2^/view). (**b**) Immunostaining of HMGB2 in WT and *Hmgb2*^−/−^ MSCs during adipogenesis. The small black boxes indicate negative controls (original magnification, x400, 0.237 mm^2^/view). (**c**) Percentage of WT MSCs positive for HMGB2. n = 3 for each time point. *p < 0.001.

Based on the microarray data, we subsequently examined endogenous *Hmgb2* expression in murine MSCs during adipogenic differentiation. Oil-red O staining showed a large increase in lipid droplets in WT MSCs on induction day 7, indicating that MSCs became mature adipocytes (Fig. 1a). During adipogenic differentiation, we found that HMGB2 was localized in the nuclei of undifferentiated WT MSCs and that *Hmgb2*^−/−^ MSCs did not exhibit HMGB2 expression (Fig. 1b). On day 3, strong HMGB2 staining was observed in WT MSCs, and the number of HMGB2-positive cells was significantly reduced on day 7 (Fig. 1c).

### Targeted disruption of *Hmgb2* in MSCs during adipogenesis

**Figure 2:**
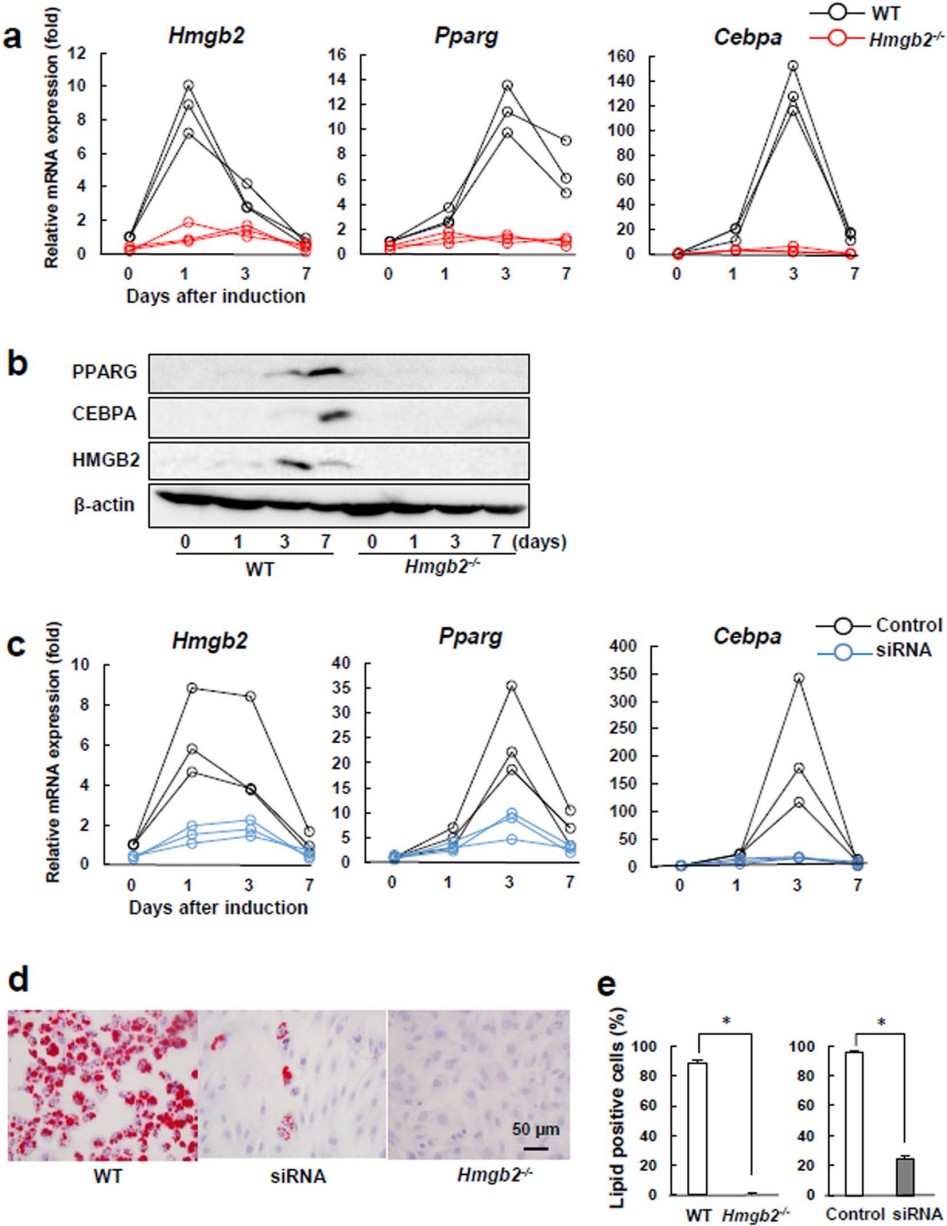
*Hmgb2* deficiency reduced MSC adipogenic differentiation. (**a**) Quantitative PCR was performed to assess the *Hmgb2, Pparg* and *Cebpa* levels in WT and *Hmgb2*^−/−^ MSCs during adipogenesis. (**b**) The expression levels in the cells at each time point in three independent experiments were determined by Western blotting of WT and *Hmgb2*^−/−^ MSCs during adipogenesis. The exposure time for HMGB2, PPARG and CEBPA was 120 sec, and the time for β-actin was 2 sec. HMGB2/β-actin and PPARG/CEBPA were detected in the different part of the each gel, respectively. Representative data from three separate experiments are shown. (**c**) Quantitative PCR in MSCs transfected with *Hmgb2* siRNA. (**d**) Oil-red O staining of WT MSCs, MSCs transfected with *Hmgb2* siRNA, and *Hmgb2*^−/−^ MSCs 7 days after adipogenic induction (original magnification, x400, 0.237 mm^2^/view). (**e**) Percentage of cells positive for lipid droplets among the WT and *Hmgb2*^−/−^ cells on day 7. n = 3 for each time point. *p < 0.001.

We assessed the endogenous expression of *Hmgb2* and adipogenesis-related markers in WT and *Hmgb2*^−/−^ MSCs. Quantitative PCR showed that the expression of *Hmgb2* peaked on induction day 1, prior to the expression of the adipogenic master transcriptional regulators *Pparg* and *Cebpa*^25^, which peaked on day 3, and then gradually decreased (Fig. 2a). Similar to the microarray data, the *Pparg*, *Cebpa* and *Hmgb2* levels were apparently decreased in *Hmgb2*^−/−^ MSCs compared with WT MSCs during adipogenesis. This HMGB2, PPARG, and CEBPA expression pattern was also reflected in the western blotting results, and bands were barely detected in *Hmgb2*^−/−^ MSCs (Fig. 2b). Full length blots and densitometry data are shown in Supplementary Fig. S1a,b, respectively.

We next sought to determine whether the attenuation of adipogenic marker expression in *Hmgb2*^−/−^ MSCs was reproducible in murine MSCs following siRNA-mediated silencing of *Hmgb2*. We observed the transcriptional expression of *Hmgb2* was reduced at approximately 63% on average after transfection of the siRNA for *Hmgb2* (Fig. 2c). Furthermore, the knockdown of *Hmgb2* also strongly suppressed the transcriptional expressions of *Pparg* and *Cebpa* afer adipogenic induction (Fig. 2c). On the other hand, the adipocytic differentiation of *Hmgb2*^−/−^ MSCs and *Hmgb2*-knockdown MSCs was significantly inhibited on day 7 when compared with that of WT MSCs (Fig. 2d,e).

### Correlation between PDGFRA and HMGB2 during MSC adipogenesis

**Figure 3:**
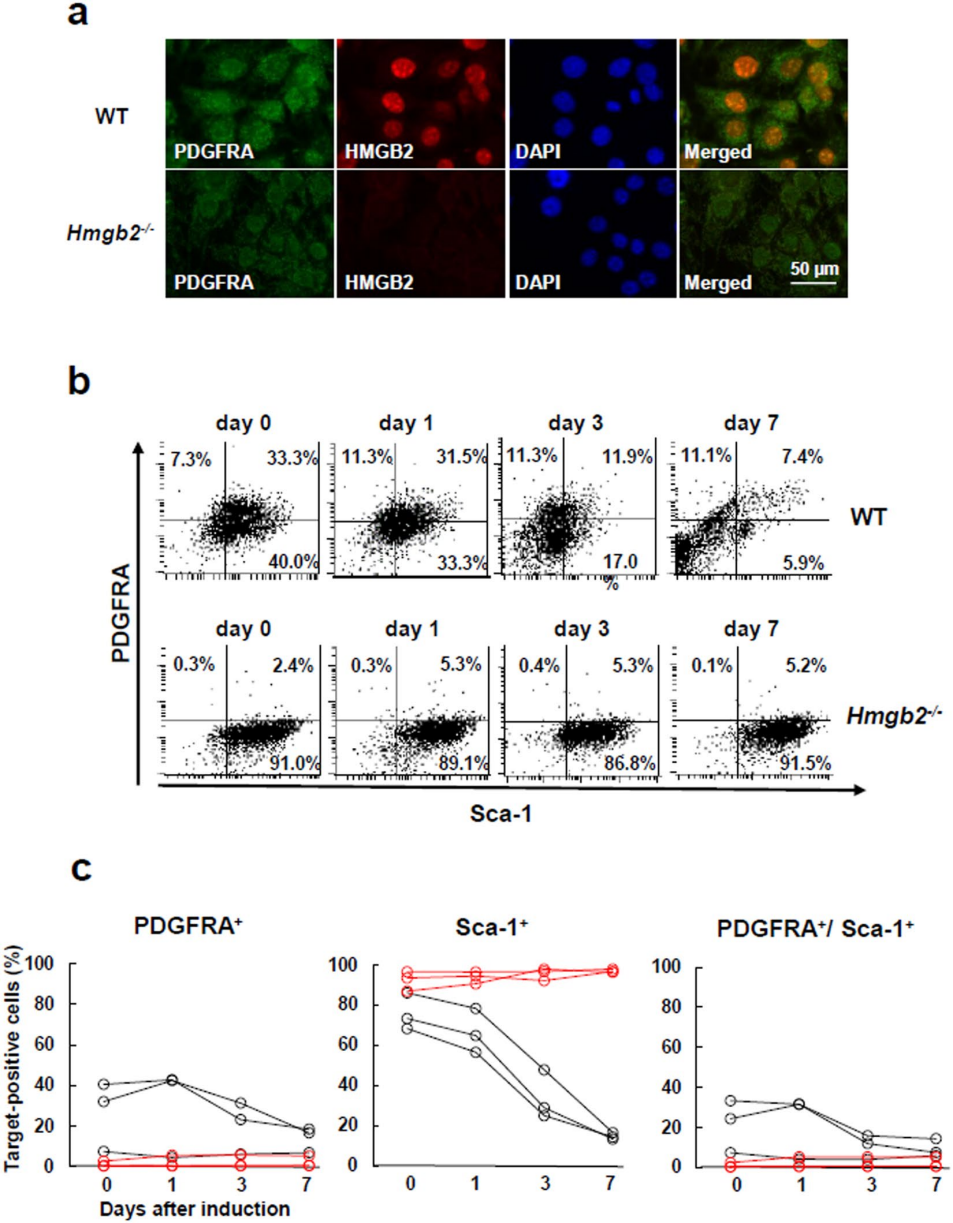
HMGB2 was co-expressed with PDGFRA in undifferentiated MSCs, and *Hmgb2* deficiency was associated with PDGFRA reduction. (**a**) Double immunofluorescence representing HMGB2 and PDGFRA expression in MSCs 1 day after adipogenic induction (original magnification, x630). (**b**) Flow cytometry analysis for the expressions of PDGFRA and Sca-1 during adipogenesis of MSCs. Representative data from three different experiments are shown. (**c**) The percentages of PDGFRA^+^, Sca-1^+^, and PDGFRA^+^/Sca-1^+^ cells in WT (black lines) and *Hmgb2*^−/−^ MSCs (red lines) are plotted. n = 3 for each time point.

Previous studies have shown that murine bone marrow-derived MSCs express PDGERA and Sca-1^8,9^, while they exhibit normal expressions of their related cell surface molecules under the deficiency of HMGB2^16^. Because HMGB2 deficiency inhibited the adipogenic differentiation of MSCs, we further evaluated the effect of the deficiency of HMGB2 on the expression of PDGERA and Sca-1 during the adipogenesis of MSCs. Immunocytochemical analysis showed that both HMGB2 and PDGFRA were expressed in the nuclei and cytoplasm in undifferentiated WT MSCs, whereas PDGFRA was barely expressed in *Hmgb2*^−/−^ MSCs (Fig. 3a). Flow cytometric analysis revealed that approximately one-third of WT MSCs expressed both PDGFRA and Sca-1 on days 0 and 1 after adipogenic induction (Fig. 3b,c and Supplementary Fig. S2). Thereafter, the frequency of the cells expressing both PDGFRA and Sca-1 were decreased, and less than 10% of the cells expressed these molecules on day 7 following adipogenic induction of WT MSCs (Fig. 3b,c, Supplementary Fig. S2a,b). In contrast, PDGFRA^+^ cells were barely detectable in *Hmgb2*^−/−^ MSCs, whereas Sca-1^+^ cells remained upon adipogenic induction (Fig. 3b,c, Supplementary Fig. S2a,b).

### HMGB2 and PDGFRA expression in a rat rotator cuff tear model

**Figure 4:**
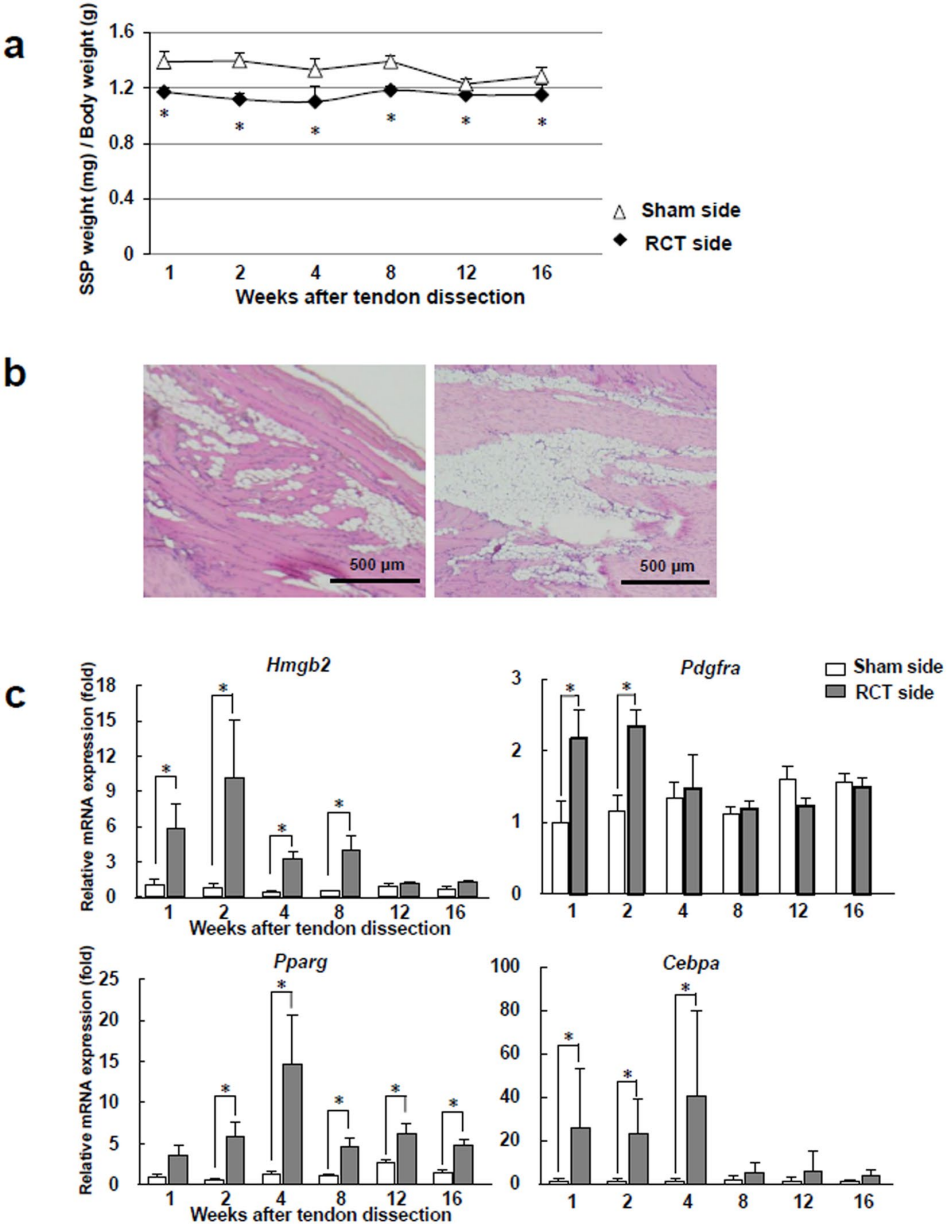
The expression of *Hmgb2* evoked and peaked prior to that of adipogenic master regulators in rat SSP muscles after tendon transection. (**a**) Ratio of the SSP muscle weight to the body weight in the rat RCT model after tendon transection. (**b**) The representative HE images of different two SSP muscle samples from RCT side 16 weeks after tendon transection (original magnification, x40, each). (**c**) Quantitative PCR analysis of SSP muscles after tendon transection. n = 6 for each time point. *p < 0.05.

To address whether the expression of HMGB2 is associated with the ectopic fat formation in skeletal muscles, we used an RCT model in rats. As reported previously^28,29^, the muscles on the RCT side were atrophic with ectopic fat infiltration 16 weeks after tendon transection (Fig. 4a,b). In supraspinatus (SSP) muscles from the RCT side, the transcriptional expressions of *Hmgb2*, *Pparg*, *Cebpa* and *Pdgfra* were significantly higher than on the sham side at early time points (Fig. 4c). Furthermore, the expression of *Hmgb2* transcript achieved a peak on 2 weeks after tendon transection, which preceded the transcriptional expressions of *Pparg* and *Cebpa*, whereas their expressions were gradually decreased thereafter.

**Figure 5:**
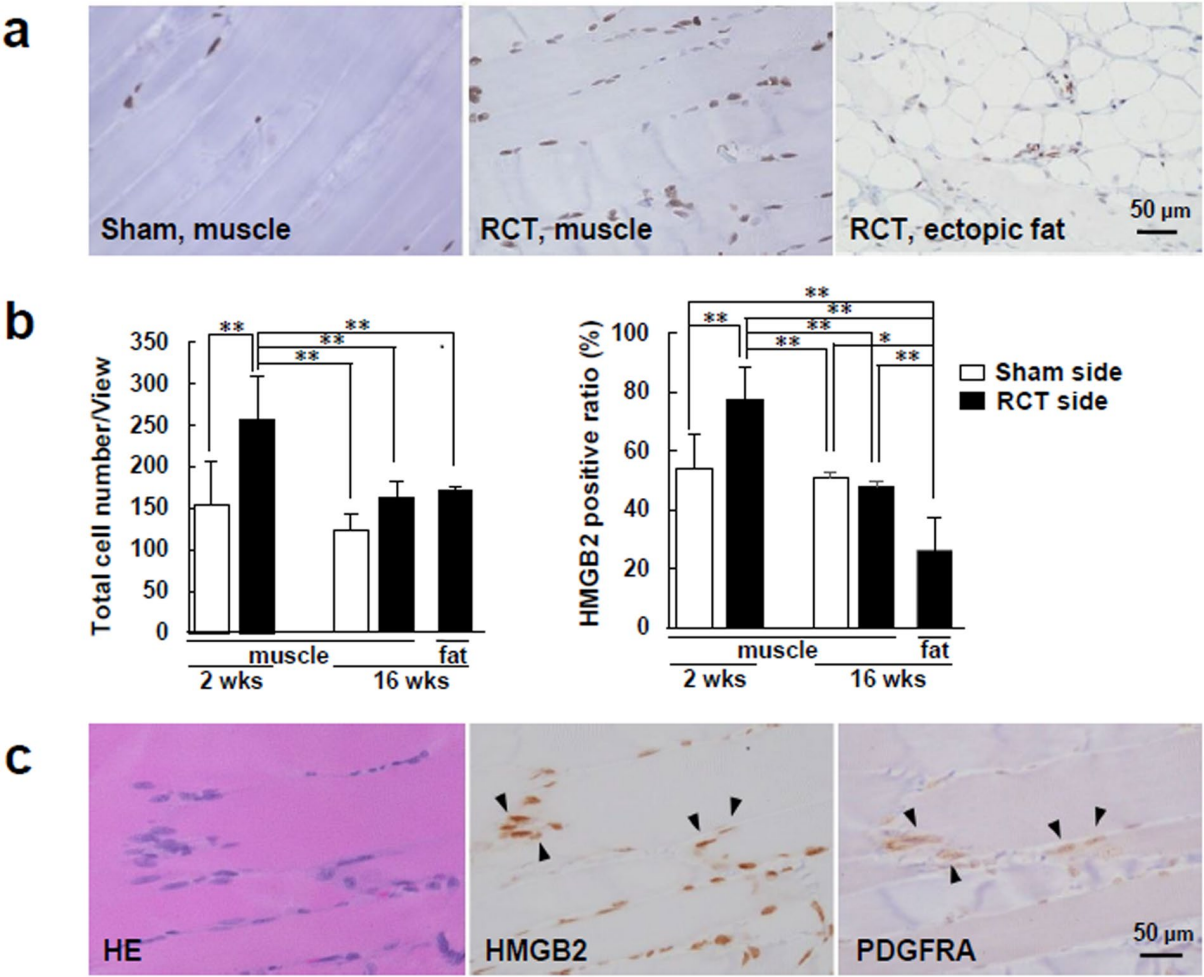
HMGB2 was abundant and partially co-expressed with PDGFRA in the intra-muscular spaces of SSP muscles from rat RCT models. (**a**) Representative images of immunostaining for HMGB2 in SSP muscles from rat RCT models (original magnification, x400). (**b**) Average total cell number and ratio of HMGB2-positive cells in the muscle and ectopic fat cells 2 and 16 weeks after tendon transection. n = 6 for each time point, 0.237 mm^2^/view. wks, weeks after tendon transection. n = 6 for each time point. *p < 0.05. **p < 0.001. (**c**) Representative images showing co-expression of HMGB2 and PDGFRA (arrowheads) in serial immunostained sections (original magnification, x400).

Immunohistochemcal analysis showed that HMGB2 was expressed in the cells in the intra-muscular spaces of SSP muscles (Fig. 5a). The average of total cell number and the ratio of HMGB2^+^ cells in the muscle belly were significantly increased on the RCT side on 2 weeks after tendon transection, but not on the sham side (Fig. 5b). In contrast, the expression of HMGB2 was markedly reduced in the ectopic fat cells on the RCT side 16 weeks after tendon transection, and this expression level was lower than that in the muscle belly on the sham side. Moreover, the cells in the intra-muscular spaces displayed a partial co-expression of HMGB2 and PDGFRA (Fig. 5c).

## Discussion

Muscle atrophy and fat infiltration are thought to be major factors limiting the success of RCT surgery and contributing to a higher rate of clinical failure^30^. The goal of this study was to identify molecular mechanisms responsible for the abnormal adipocyte differentiation and fat infiltration observed in the pathogenesis of RCT disease. Microarray analysis of bone marrow-derived MSCs from WT and *Hmgb2*−/− mice prompted the idea that HMGB2, which is highly expressed in MSCs^16^, may be important for regulating adipogenesis. Additionally, we verified that both MSCs derived from *Hmgb2*^−/−^ mice and *Hmgb2*-knockdown MSCs with siRNA-mediated silencing derived from WT mice lost the ability to differentiate into adipocytes *in vitro*.

PPARG and CEBPA are major transcription factors in adipogenesis that cooperatively induce the expression of adipocyte-specific genes and are involved in the switch between proliferation and differentiation in cells^26,31^. Our *in vitro* data showed that *Pparg* and *Cebpa* were among the genes that were strongly downregulated by *Hmgb2* suppression. Furthermore, we found that endogenous *Hmgb2* expression in WT MSCs increased earlier than that of *Pparg* and *Cebpa* during adipogenesis. Studies in adipogenic cell lines have shown that hormonal induction of differentiation is rapidly induced by CEBPB and CEBPD, which begin to decrease coincident with a rise in CEBPA and PPARG^26^. CEBPB is a direct target gene of HMGB2 in senescent cells, and the knockdown of HMGB2 decreases *Cebpb* gene expression^32^; thus, HMGB2 may be an upstream factor regulating PPARG and CEBPA in MSC adipogenesis (Supplementary Fig. S3).

The HMGB2 mRNA and protein levels were enhanced in the early stage of adipogenesis and then gradually decreased when MSCs differentiated into mature adipocytes. This expression pattern is unique because HMGB2 expression, which is robust in undifferentiated MSCs, gradually decreases during other differentiation programs, such as myogenesis, chondrogenesis, and osteogenesis^16,20^. In our animal models, the transcriptional expression of HMGB2 was enhanced at the intra-muscular spaces at early stage after tendon transection, and then was barely detectable in ectopic fat cells at late stage. These phenomena led us to hypothesize that HMGB2 is prerequisite for the differentiation of MSCs during adipogenesis, myogenesis, chondrogenesis, and osteogenesis, while it is dispensable for the maintenance of the features of MSCs and their differentiated cell type. Collectively, these results suggest that HMGB2 is transiently expressed upon adipogenic induction, and that promotes the developmental process from MSCs into mature adipocytes or ectopic fat cells for the fat infiltration in skeletal muscle.

We have previously shown that MSCs exhibit normal cell surface expressions of CD44, CD29, and Sca-1 with lack of CD45, CD31, and CD34 under the deficiency of HMGB2, while they have an ability to differentiate into chondrocyte and osteocytes^16,33^. In contrast, *Hmgb2*^−/−^ MSCs abnormally differentiate into adipocytes in terms of cell surface expressions of PDGFRA and Sca-1. These results suggest that HMGB2 is prerequisite for the differentiation of MSCs into adipocytes probably due to enhancement of the adipogenic signaling cascades.

The appearance of ectopic fat and fibrosis in skeletal muscles has been reported to be common in PDGFRA-positive cells localized in intra-muscular spaces^5–7^. The cells in which these phenomena originate were identified as MSCs rather than satellite cells or pericytes in the intra-muscular spaces, which are negative for PDGFRA. Based on these findings, we initially examined the expression of HMGB2 and PDGFRA in an *in vitro* culture model and found that both proteins were co-expressed in undifferentiated murine MSCs, followed by elimination after adipogenic maturation. Importantly, PDGFRA was notably decreased in *Hmgb2*^−/−^ MSCs, which never differentiated into adipocytes, although Sca-1 was unaffected. These findings are compatible with a previous report showing that *Pdgfra*^−/−^ murine MSCs cannot differentiate into adipocytes^5^. Thus HMGB2-PDGFRA cascade may play a key role in maintaining the pluripotency of MSCs toward the adipogenic lineage. In this context, a recent study showed that correcting the expression of HMGB2 and PDGFRA may be a potential therapeutic strategy for restoring the dysregulation of lipid metabolism in obese women with polycystic ovarian syndrome^34^.

A recent study showed that the population of PDGFRA-positive MSCs increased rapidly in murine SSP muscles after rotator cuff transection, and fat infiltration was reduced by PDGFR signal inhibition^35^. In our rat model, *Pdgfra* was enhanced after tendon transection, and the population of HMGB2-positive cells also increased in the SSP muscles with RCT. The co-expression of HMGB2 and PDGFRA in intra-muscular spaces was consistent with our flow cytometry data, which showed that approximately 40% of WT murine MSCs were positive for PDGFRA, although those cells were barely detectable in *Hmgb2*^−/−^. These findings indicate that HMGB2-PDGFRA-positive cells may be candidates for regulating adipogenesis as well as fat infiltration into skeletal muscles and suggest that these cells might be the origin of ectopic fat cells in ruptured rotator cuffs.

Pathway analysis of the DNA microarray suggests that Wnt signaling pathway is activated under the deficiency of HMGB2. Previous studies have shown that Wnt signaling has a key role to control the differentiation of MSCs during osteogenesis and myogenesis, and suppressing adipogenesis^27,36^. Furthermore, it has been reported that the changes in the activation status of Wnt signaling are observed in musculoskeletal system in response to the mechanical stimulations such as compression, tension and shearing^37,38^. On the other hand, the fat infiltration has been shown to suppress Wnt signal to enhance the expressions of *Pparg* and *Cebpa* in a rat RCT model^38^. In addition, it has been shown that the chronic tendon rupture, i.e. prolonged unloading state of muscles, often results in the highly degenerated muscles^39^, while continuous resistance training ameliorates muscle fibrosis and atrophy in aged mice mediated through the activation of Wnt signal^40^. Another studies demonstrated that mechanical stretching of muscle directly activates Wnt signaling to inhibit myoblast differentiation into adipocytes^41^. The interaction of HMGB2 with LEF1 enhances the Wnt/β-catenin pathway in articular cartilage^15^, and one possible mechanism explaining the observed modulation of LEF1-dependent transactivation by HMGB2 is that differential interactions between HMGB2 and nuclear factors affect the transcription of adipogenic genes containing LEF1-responsive elements. These findings may help elucidate the involvement of HMGB2 in the switch of MSCs to mature adipocytes or ectopic fat cells formation in the muscles.

In conclusion, we report that HMGB2 play a crucial role in regulating the adipogenesis of MSCs and fat infiltration into skeletal muscles following rotator cuff tear through the cascade of HMGB2-PDGFRA. In future studies, a better understanding of the nature of HMGB2 in the adipogenesis of MSCs may open new avenues for exploring therapeutic strategies to mediate the neutralization of its function for the ectopic fat formation in skeletal muscles with chronic tendon tears.

## Methods

All animal experiments were conducted in compliance with a protocol approved by the Institutional Animal Care and Use Committee of Miyazaki University (no. 2015-518-4) and The Scripps Research Institute (no. 09-0130-3).

### Preparation of murine MSCs and culture

Bone marrow-derived MSCs were prepared from the tibias and femurs of 6- to 8-week-old C57BL/6 J WT and *Hmgb2*^−/−^ mice without sexual selection as previously reported^16,42,43^. At each passage during culturing the cells, cells were stained with phycoerythrin-conjugatedm mAbs to CD45, CD44 (BD PharMingen, Franklin Lakes, NJ, USA), CD34 (Biolegend, San Diego, CA, USA), Sca-1, CD29, and CD31 (eBioscience, San Diego, CA, USA) and analyzed by flow cytometry to determine the phenotype of the cells^16^. The ability of the cells to undergo chondrogenesis and osteogenesis was assessed by quantitative PCR for *Col2a1*, *Agc1*, *Col10a1, Alpl, Ibsp, Bglap, and Runx2*, and confirmed by Safranin O and alizarin red S staining^16^. The adipogenic induction of MSCs was examined using the Poietics Mesenchymal Stem Cell Differentiation System according to the manufacturer’s instructions (Lonza, PT-3004, Basel, Switzerland), and the cells were harvested 0, 1, 3 and 7 days after induction in three independent experiments for quantitative PCR, western blotting, Oil-red O staining, immunocytochemistry and flow cytometry analyses (n = 3 for each time point).

### RCT model in rats

Thirty-six male Sprague-Dawley rats (16 weeks; 450 to 550 g body weight) underwent massive surgical rotator cuff transection as described previously^28,44^. After the supraspinatus and infraspinatus tendons were transected at the transition between muscles and tendons, the remaining tendons on the bone side were removed. To generate a sham surgery control, the left deltoid muscles of the rats were also split. Surgery and resuscitation were performed on a heat board and under heat lamps. After surgery, buprenorphine (0.05 mg/kg) was administered subcutaneously to control pain^28^, and animals were housed in normal conditions. Six rats were euthanized with intra-peritoneal overdose injection of sodium pentobarbital at each time point (1, 2, 4, 8, 12 and 16 weeks) after tendon transection, and SSP muscles were harvested for quantitative PCR and immunohistochemistry (n = 6 for each time point). The average ratio of the SSP muscle weight to the body weight was calculated at each time point and compared between the RCT and sham sides. We did not observed the mortality or complications of animals before the anticipated end point.

### Microarray analysis of murine MSCs

RNA was isolated from WT and *Hmgb2*^−/−^ MSCs (n = 2 each due to the financial limitation), and cRNA was prepared as described previously^16^. Fifteen micrograms of fragmented cRNA from each sample was hybridized to a pre-equilibrated Affymetrix chip (Affymetrix Inc., MoGene-1_0-st-v1 Santa Clara, CA, USA), which was then washed, stained, and scanned in an HP ChipScanner (Affymetrix Inc.) as described previously^45^. Data normalization was performed using GeneSpring (Agilent Technologies, Santa Clara, CA, USA). All entities (number: 28,853) were filtered based on expression ranging from the 20.0 to 100.0th percentile according to the raw data (number: 22,795). The entities list was made from transcripts showing an average expression level in *Hmgb2*^−/−^ per average expression level in WT ≥ 2 or ≤0.5. The overlapping rates of entities randomly selected from the entities list (n = 426) and factors in a pathway listed in GeneSpring were calculated as p-values by hypergeometric test using GeneSpring. p < 0.05 was considered statistically significant. The pathways in which the overlapping factors were increased or decreased in *Hmgb2*^−/−^ MSCs were considered activated or inhibited in *Hmgb2*^−/−^ MSCs, respectively.

### Quantitative PCR

Total RNA was extracted and reverse transcribed into cDNA as described previously^46^. Gene expression was analyzed using SYBR Green and a Step One Plus Real-time PCR system (Thermo Fisher Scientific, Waltham, MA, USA). All samples were assayed in triplicate, and the CT values were averaged. GAPDH was employed as the internal control for rat muscles, and β-actin was used as the control for murine cells. The sequences of the primers used in the study are shown in Supplementary Table S1.

### Gene knockdown of *Hmgb2* in murine MSCs

Murine MSCs were cultured in six-well plates (2 × 10^5^ per well) and transfected with 100 mM small interfering RNA (siRNA) targeting HMGB2 (Integrated DNA Technologies, MMC.RNAI.N008252.3_2 nm, Coralville, IA, USA)^15^ or the negative control (Integrated DNA Technologies, DS Scrambled-Neg universal negative control duplex) using Lipofectamine RNAi MAX reagent (Invitrogen, Carlsbad, CA, USA) according to the manufacturer’s instructions. Adipogenic induction of MSCs was initiated 24 h after transfection with siRNA or negative control in three independent experiments as described above. Only the product of Integrated DNA Technologies without fluorescent labeling worked.

### Oil-red O staining

To stain adipocytes, the cells were fixed in 10% formalin, rinsed in water and 60% isopropanol, stained with Oil-red O (Wako, Osaka, Japan) in 60% isopropanol, and then rinsed in PBS. Hematoxylin was used for counter staining.

### Immunohistochemistry detection of HMGB2 and PDGFRA

Rat SSP muscle samples were fixed using 4% paraformaldehyde/PBS, paraffin-embedded, and cut into 3-µm-thick sections. After deparaffinization, the slides were autoclaved at 120 °C for 15 min in 10 mM citrate buffer (pH 6.0) to detect HMGB2 and in 10 mM Tris-EDTA buffer (pH 9.0) to detect PDGFRA for antigen retrieval. After inhibition of endogenous peroxidase activity with 3% H_2_O_2_ in methanol for 15 min, the sections were preincubated for 1 h with 500 µg/ml normal goat IgG or 5 µg/ml normal rabbit IgG to block non-specific binding of HMGB2 and PDGFRA antibodies, respectively. The slides were subsequently incubated overnight at 4 °C with rabbit anti-HMGB2 (1:500 dilution, Abcam, ab11973, Cambridge, UK) or goat anti-PDGFRA (5 µg/ml, R&D, AF-307-NA, Minneapolis, MN, USA) antibodies, and normal rabbit IgG and normal goat IgG were employed as the respective negative controls. After washing with 0.075% Brij 35 (Sigma-Aldrich, St. Louis, MO, USA) in PBS, the slides were reacted with an HRP-conjugated goat anti-rabbit or HRP-conjugated rabbit anti-goat secondary antibody for 1 h. The HRP sites were visualized with DAB, and hematoxylin was used for nuclear counterstaining.

For immunocytochemistry, cultured cells were fixed in 4% paraformaldehyde/PBS and treated with Histo VT One solution (Nakarai, Kyoto, Japan) for 20 min at 60 °C. Blocking of non-specific binding of antibodies was performed as described above. The same primary HMGB2 and PDGFRA antibodies used to probe rat muscles were incubated with cells for 1 h at room temperature. An HRP-linked goat anti-rabbit secondary antibody was employed for AEC visualization of HMGB2. Alexa-488-conjugated goat anti-rabbit and Alexa-594-conjugated rabbit anti-goat secondary antibodies were subsequently added for immunofluorescence detection, followed by incubation for 1 h. DAPI (Invitrogen, D1306) was used for nuclear counterstaining.

### Cell counting

During MSC adipogenesis, Oil-red O-positive cells and AEC-positive cells were manually counted as adipocytes and HMGB2-positive cells, respectively, in 10 fields of view (magnification, x400, 0.237 mm^2^/view) for each condition. In SSP muscles from rat models, DAB-positive cells were manually counted as HMGB2-positive cells in different 5 views containing only muscle or fat tissue (magnification, x400, 0.237 mm^2^/view). The views were randomly selected in different regions not to be next to each other. The ratio of Oil-red O-positive cells or HMGB2-positive cells/total cells, including hematoxylin-positive cells, was calculated.

### Western blotting

Cultured cells were lysed in RIPA buffer (Sigma-Aldrich). A total of 20 µg of each lysate was separated on 12% (w/v) Tris-glycine SDS-polyacrylamide gel electrophoresis gels and transferred to PVDF membranes (Invitrogen). After the membranes were blocked in 5% skim milk, they were incubated overnight at 4 °C with rabbit anti-HMGB2 antibody (3 µg/ml, Abcam, ab6782), rabbit anti-CEBPA antibody (2 µg/ml, Abcam, ab15048), rabbit anti-PPARG antibody (2 µg/ml, Abcam, ab45036) and mouse anti-β-actin antibody (1:50,000 dilution, Sigma-Aldrich, A2228). After washing with TBS-T, the membranes were incubated for 1 h with HRP-conjugated anti-rabbit secondary antibody for targets and with HRP-conjugated goat anti-mouse secondary antibody for β-actin. Then, the protein bands were visualized by chemiluminescence using an ImageQuant LAS4000 imaging system (GE Healthcare, Japan).

### Flow cytometry

Cells were suspended in ice-cold Fc Block (anti-CD16/CD32 mAb; 1 mg/ml, BD Biosciences, Franklin Lakes, NJ, USA) at 5 × 10^7^ cells/ml and then stained with APC-conjugated PDGFRA (eBioscience, 17-1401-81, San Diego, CA, USA) and FITC-conjugated Sca-1 (eBioscience, 11–5981) antibodies for 30 min on ice. Fluorescence was analyzed using a FACSCalibur flow cytometer and CELLQuest Software (both from BD Biosciences). PI (Sigma-Aldrich, P4170) fluorescence was measured to define a live cell gate.

### Statistical analysis

Statistical analyses were analyzed using SPSS 21 (IBM, Armonk, NY, USA). Differences were assessed using one-way ANOVA in Fig. 1c. Because the factors did not exhibit a normal distribution based on a Shapiro-Wilk test, the differences shown in Figs 2d and 4a,c,d were assessed using Wilcoxon signed-rank tests. These results are presented as means ± SD, and p < 0.05 was considered statistically significant. In Figs 2a,c and 3c, and Supplementary Fig. S1b, all the values were plotted and connected to show the range of each factor (n = 3) at each time point.

### Data availability

The datasets generated and analyzed in the current study are available from the corresponding author upon reasonable request.

## Electronic supplementary material

Supplementary Information
