# Author Correction: HMGB2 is a novel adipogenic factor that regulates ectopic fat infiltration in skeletal muscles

**DOI:** 10.1038/s41598-020-61550-w

**Published:** 2020-03-10

**Authors:** Deokcheol Lee, Noboru Taniguchi, Katsuaki Sato, Narantsog Choijookhuu, Yoshitaka Hishikawa, Hiroaki Kataoka, Hidetaka Morinaga, Martin Lotz, Etsuo Chosa

**Affiliations:** 1 Department of Orthopaedic Surgery, University of Miyazaki, 5200 Kihara, Kiyotake, Miyazaki 889-1692 Japan; 2 Institute of Medical Science, Tokyo Medical University, 6-1-1 Shinjuku, Shinjuku-ku, Tokyo 160-8402 Japan; 3 Division of Immunology, Department of Infectious Diseases, University of Miyazaki, 5200 Kihara, Kiyotake, Miyazaki 889-1692 Japan; 4 Department of Anatomy, Histochemistry and Cell Biology, University of Miyazaki, 5200 Kihara, Kiyotake, Miyazaki 889-1692 Japan; 5 Section of Oncopathology and Regenerative Biology, Department of Pathology, University of Miyazaki, 5200 Kihara, Kiyotake, Miyazaki 889-1692 Japan; 6 Department of Internal Medicine and Bioregulatory Science, Graduate School of Medical Sciences, Kyushu University, 3-1-1 Maidashi, Higashi, Fukuoka 812-8582 Japan; 7 Department of Molecular Medicine, The Scripps Research Institute, 10550 North Torrey Pines Road, La Jolla, CA 92037 USA

Correction to: *Scientific Reports* 10.1038/s41598-018-28023-7, published online 25 June 2018

The original version of this Article contained an error in Affiliation 6, which was incorrectly given as ‘Department of Internal Medicine and Bioregulatory Science, Graduate School of Medical Sciences, 3-1-1 Maidashi, Higashi, Fukuoka, 812-8582, Japan’. The correct affiliation is listed below:

Department of Internal Medicine and Bioregulatory Science, Graduate School of Medical Sciences, Kyushu University, 3-1-1 Maidashi, Higashi, Fukuoka, 812-8582, Japan.

Additionally, in the Supplementary Information file originally published with this Article, Affiliation 2 was incorrectly given as ‘Department of Medical Science, Tokyo Medical University, 6-1-1 Shinjuku, Shinjuku, Tokyo 160-8402, Japan’. The correct affiliation is listed below:

Institute of Medical Science, Tokyo Medical University, 6-1-1 Shinjuku, Shinjuku-ku, Tokyo, 160-8402, Japan

These errors have now been corrected in the HTML and PDF versions of the article, alongside the Supplementary information file.

