# Supplementary material for: HMGB2 is a novel adipogenic factor that regulates ectopic fat infiltration in skeletal muscles
Source: Sci Rep. 2018 Jun 25;8:9601. doi: 10.1038/s41598-018-28023-7 (PMC6018498; doi:10.1038/s41598-018-28023-7)
Supplement: Supplementary file 1 — Supplementary Information [file 41598_2018_28023_MOESM1_ESM.pdf]

HMGB2 is a novel adipogenic factor that regulates ectopic fat infiltration in skeletal muscles

Deokcheol Lee[1], Noboru Taniguchi[1, 2,*], Katsuaki Sato[3,*], Narantsog Choijookhuu[4], Yoshitaka Hishikawa[4], Hiroaki Kataoka[5], Hidetaka Morinaga[6], Martin Lotz[7] & Etsuo Chosa[1]

[1]Department of Orthopaedic Surgery, University of Miyazaki, 5200 Kihara, Kiyotake, Miyazaki 889-1692, Japan
[2]Institute of Medical Science, Tokyo Medical University, 6-1-1 Shinjuku, Shinjuku-ku, Tokyo, 160-8402, Japan
[3]Division of Immunology, Department of Infectious Diseases, University of Miyazaki, 5200 Kihara, Kiyotake, Miyazaki 889-1692, Japan
[4]Department of Anatomy, Histochemistry and Cell Biology, University of Miyazaki, 5200 Kihara, Kiyotake, Miyazaki 889-1692, Japan
[5]Section of Oncopathology and Regenerative Biology, Department of Pathology, University of Miyazaki, 5200 Kihara, Kiyotake, Miyazaki 889-1692, Japan
[6]Department of Internal Medicine and Bioregulatory Science, Graduate School of Medical Sciences, Kyushu University, 3-1-1 Maidashi, Higashi, Fukuoka 812-8582, Japan
[7]Department of Molecular Medicine, The Scripps Research Institute, 10550 North Torrey Pines Road, La Jolla, CA 92037, USA

Correspondence should be addressed to: Noboru Taniguchi, MD, PhD,
Department of Orthopaedic Surgery,University of Miyazaki, 5200 Kihara, Kiyotake, Miyazaki 889-1692, Japan
Phone: +81-985-85-0986, Fax: +81-985-84-2931,
e-mail: nobutanigu@gmail.com

[*]Corresponding author: Katsuaki Sato, PhD
Division of Immunology, Department of Infectious Diseases, University of Miyazaki, Miyazaki
5200 Kihara, Kiyotake, Miyazaki 889-1692, Japan
Tel.: +81-985-85-9815, Fax: +81-985-84-9899

Email: katsuaki_sato@med.miyazaki-u.ac.jp

Table S1. Sequences of the primers used in quantitative PCR.

Murine Gene	Sequences	Rattus Gene	Sequences
Hmgb2	F: TCCTGGTAGGCCAACAGGCT	***Hmgb2***	F: TCCTGGTAGGCCAACAGGCT
	R: AGCTAATGTTGAGCTGCACTTG		R: AGCTAATGTTGAGCTGCACTTG
Pparg	F: GAAAGACAACGGACAAATCACC	***Pparg***	F: CGAAGAACCATCCGATTGAAGC
	R: GGGGGTGATATGTTTGAACTTG		R: CCAAACCTGATGGCATTGTGA
Cebpa	F: CAAGAACAGCAACGAGTACCG	***Cebpa***	F: TGACCAGTGACAATGACCGC
	R: GTCACTGGTCAACTCCAGCAC		R: GCGACCCTAAACCATCCTCC
β-actin	F: GAGCTATGAGCTGCCTGACG	***Pdgfra***	F: AAACGGTGAGGAACTCGACTC
	R: AGTTTCATGGATGCCACAGG		R: GAAACTTTGTGCTGCTCTGC
		Gapdh	F: CCGCATCTTCTTGTGCAGTG
			R: GAGAAGGCAGCCCTGGTAAC

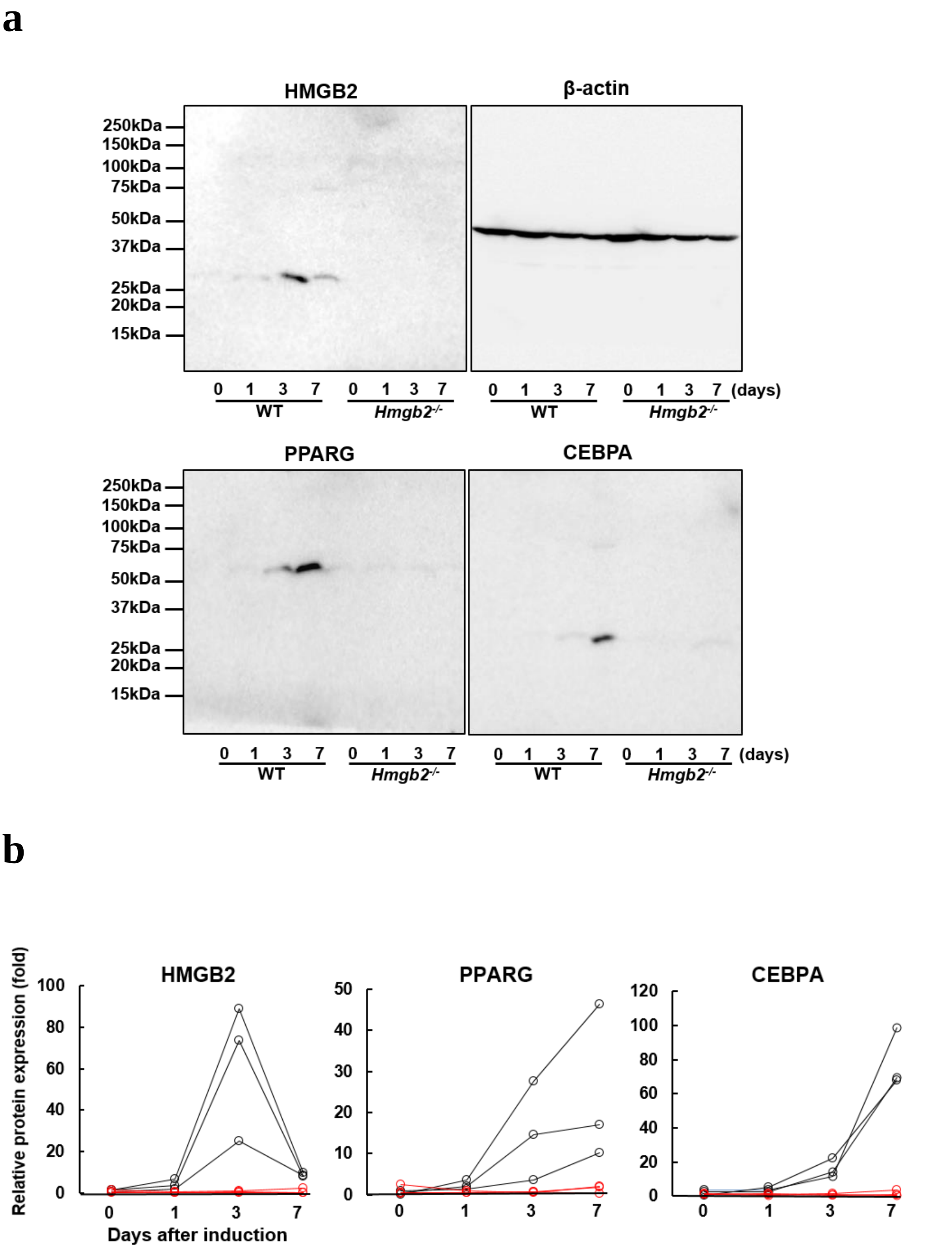

Figure S1. Western blotting of WT and *Hmgb2*$^{-/-}$ MSCs for HMGB2, PPARG, and CEBPA during adipogenesis. (a) Full length of the blotting are shown. The exposure time for HMGB2, PPARG and CEBPA was 120 sec, and the time for β-actin was 2 sec. HMGB2/β-actin and PPARG/CEBPA were detected in the different part of the each gel, respectively.

Representative data from three separate experiments are shown. (b) The relative expression levels of the targets compared to internal control in WT MSCs (black line) and *Hmgb2*$^{--/}$ MSCs (red line). The values were quantified by assessing the density of bands using Image J (National Institutes of Health, Bethesda, MD, USA), and all the values were plotted and connected to show the range of each factor (n = 3) at each time point.

a

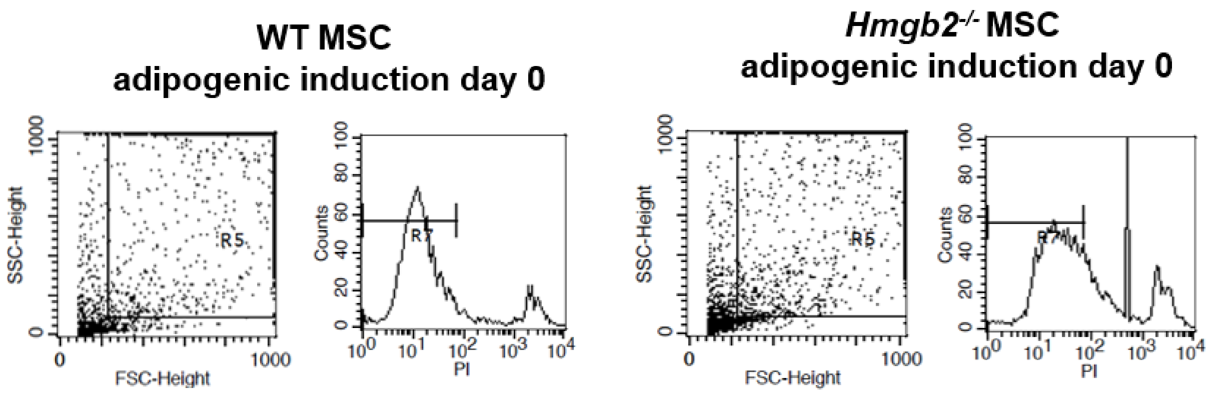

b

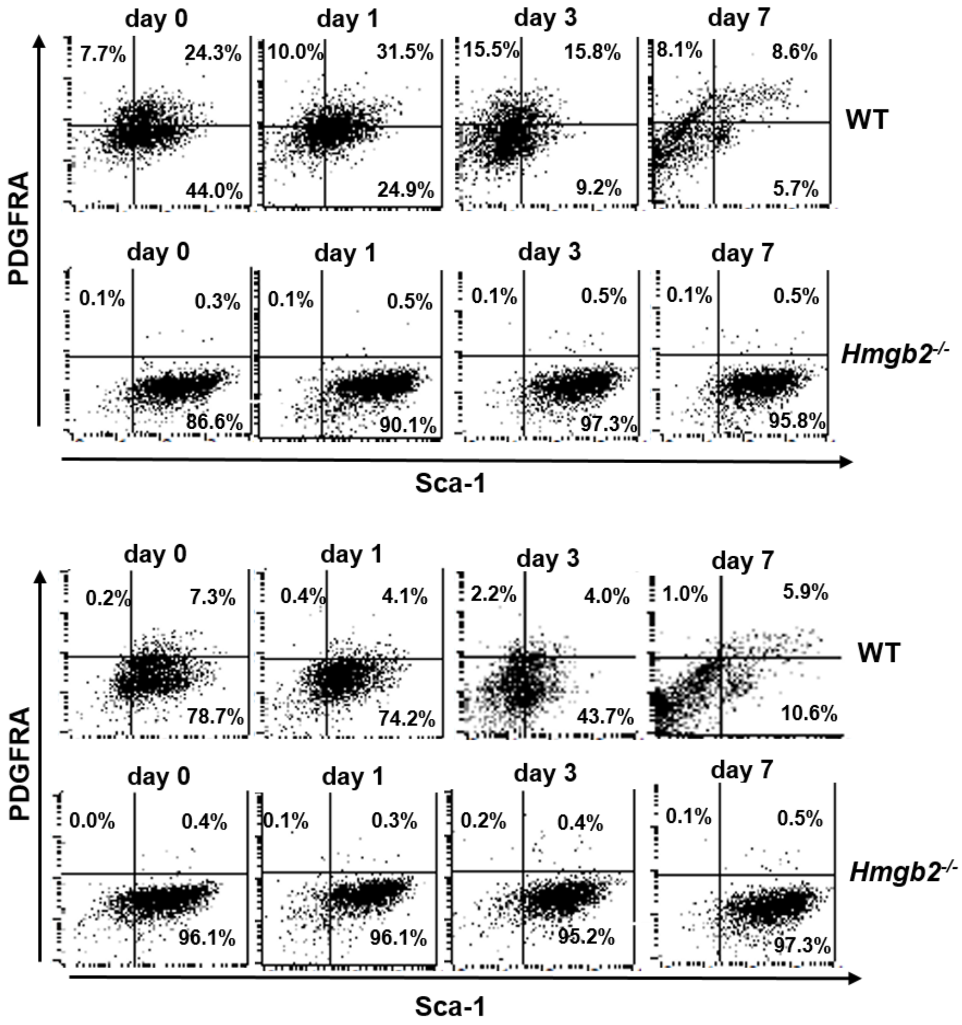

Figure S2. Flow cytometry analysis for the expressions of PDGFRA and Sca-1 during adipogenesis of MSCs. (a) The cells were analyzed in the indicated sequential gates for

forward scatter-area (FSC-A)/side scatter area (SSC-A) (R5), and propidium iodide (PI) to exclude dead cells (R7). The representative percentages for (R5) and (7) on adipogenic induction day 0 were 93.5 % and 86.7 % in WT MSC cells, whereas they were 94.2% and 69.1 % in *Hmgb2*$^{-/-}$ MSC cells. (b) The data from three different experiments except for the representative data (Fig. 3b) are shown.

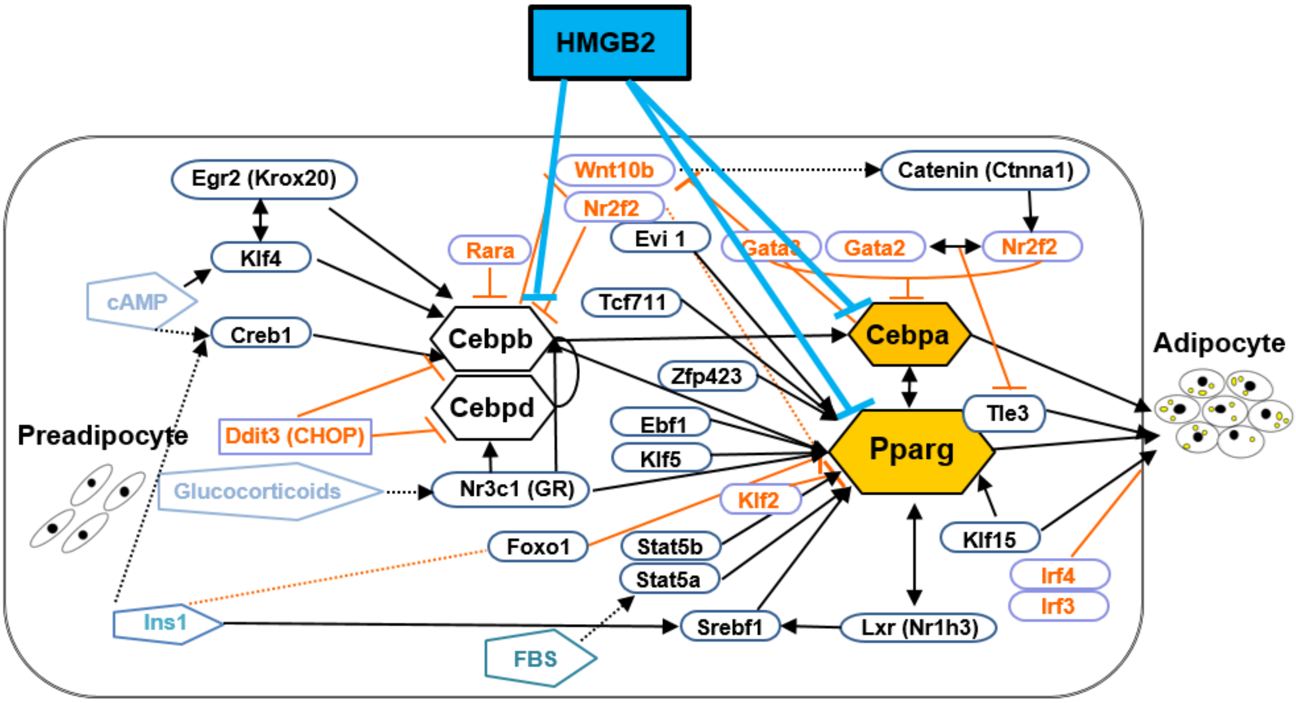

Figure S3. The adipogenesis pathway was suppressed in *Hmgb2*$^{-/-}$ MSCs. In *Hmgb2*$^{-/-}$ MSCs, adipogenesis-related pathways, such as the PPAR signaling pathway and white fat cell differentiation-related pathways were suppressed.
